# Champ4life Study Protocol: A One-Year Randomized Controlled Trial of a Lifestyle Intervention for Inactive Former Elite Athletes with Overweight/Obesity

**DOI:** 10.3390/nu12020286

**Published:** 2020-01-21

**Authors:** Analiza M. Silva, Catarina L. Nunes, Catarina N. Matias, Filipe Jesus, Rúben Francisco, Miguel Cardoso, Inês Santos, Eliana V. Carraça, Marlene N. Silva, Luís B. Sardinha, Paulo Martins, Cláudia S. Minderico

**Affiliations:** 1 Exercise and Health Laboratory, CIPER, Faculdade Motricidade Humana, Universidade Lisboa, Estrada da Costa, 1499-688 Cruz-Quebrada, Portugal; catarinanunes@fmh.ulisboa.pt (C.L.N.); cmatias@fmh.ulisboa.pt (C.N.M.); fastj96@gmail.com (F.J.); ruben92francisco@gmail.com (R.F.); m73cardoso@gmail.com (M.C.); isantos@fmh.ulisboa.pt (I.S.); ecarraca@fmh.ulisboa.pt (E.V.C.); mnsilva@fmh.ulisboa.pt (M.N.S.); lsardinha@fmh.ulisboa.pt (L.B.S.); cminderico@gmail.com (C.S.M.); 2 Physiology and Biochemistry Laboratory, CIPER, Faculdade Motricidade Humana, Universidade Lisboa, Estrada da Costa, 1499-002 Cruz-Quebrada, Portugal; 3 Laboratório de Nutrição, Faculdade de Medicina, Universidade de Lisboa, 1649-028 Lisboa, Portugal; 4 Faculdade de Educação Física e Desporto, Universidade Lusófona de Humanidades e Tecnologias, 1749-024 Lisboa, Portugal; 5 Laboratory of Sport Psychology, Faculdade de Motricidade Humana da Universidade de Lisboa, 1499-002 Cruz-Quebrada, Portugal; pmartins@fmh.ulisboa.pt

**Keywords:** obesity, former athletes, behavior change, sedentary behavior, physical activity, energy balance regulation

## Abstract

Preventive and educational programs directed to former elite athletes in the areas of healthy living are required. This is particularly relevant as obesity and health-related problems are observed in retired athletes, especially in those whose current levels of physical activity are below the recommendations. During their sports career, elite athletes are supported by a multidisciplinary team; upon retirement, no support is provided for the transition to a different lifestyle. So far, no program has been implemented to promote sustained healthy lifestyle behaviors in the post-career transition and evidence is lacking for such an intervention. Firstly, we aim to determine if Champ4life, a 1-year lifestyle intervention targeting inactive former athletes with overweight and obesity, is effective for reducing total and abdominal fat. Secondly, our purpose is to assess the effectiveness of the intervention on the levels of physical activity and sedentary behavior, resting energy expenditure, cardio-metabolic markers, physical fitness, energy balance components, eating self-regulation markers, and quality of life over 12 months. Champ4life is an evidence- and theory-based program using a randomized control trial design (intervention vs. control group) that will be conducted on 94 inactive former elite athletes with overweight and obesity. The first four months of the Champ4Life program include a nutritional appointment and 12 weekly, 90-min sessions. Classroom sessions seek to provide participants with key information and a toolbox of behavior change techniques to initiate and sustain long-term lifestyle changes. Participants will undergo baseline, 4-month, and 12-month measurements of body composition (primary outcomes), resting energy expenditure, physical fitness, metabolic markers, energy balance related-markers, and quality of life (secondary outcome). This trial will provide evidence on the effectiveness of the Champ4life program, a pioneer lifestyle intervention for retired athletes, offering tools for sustained changes in physical activity, sedentary behavior and diet, aiming to improve body composition and overall health-related markers.

## 1. Introduction

Athletes are required to match their energy demands with an adequate energy intake to ensure good performance during their sports careers [1,2]. However, the transition to post-career life is inevitable and adopting new strategies that can accommodate their new (lower) energy requirements are crucial to avoid undesired weight gain, a difficult challenge that athletes are faced with [3].

Obesity is caused by an alteration in the energy balance status caused by energy intake (EI) that surpasses the energy expenditure (EE). It is expected that after finishing their competitive careers in sport, athletes dramatically reduce their EE, which puts them at increased risk of developing obesity, particularly in those who became inactive upon retirement [4]. Stubbs et al. [5] observed that reducing physical activity (PA) level does not necessarily induce an equivalent reduction in EI, possibly resulting in a positive energy balance and subsequent weight gain, thus increasing the risk of developing obesity-related adverse health effects. Recently, retired football linemen with higher body mass indexes were found to have a greater incidence of metabolic syndrome, dyslipidemia, and elevated fasting plasma glucose compared to non-linemen [6]. Increased blood pressure, triglycerides, and dyslipidemia were observed in retired National Football League (NFL) players compared with age-matched controls [7]. A recent systematic review underlined that retired athletes with an elevated body mass index had an increased prevalence and more severe risk factors for cardiovascular disease [3]. Hence, besides an early cardiovascular risk factor screening, maintenance of sufficient PA levels in early retirement has been advocated [6,8,9,10]. Prior research on former athletes showed that many did not maintain an exercise routine when they finished their competitive athletic careers [4] and also that health-related risks were particularly present in those exposed to a sedentary lifestyle [11].

In Portugal, based on self-reported weight and height, the prevalence of overweight/obesity in former elite athletes is high (~50%) [12], with most athletes report difficulties in managing the end of their career [13], likely due to the lack of support in this transition period. Literature is abundant on the effect of lifestyle interventions targeting individuals with overweight and obesity [14,15,16]. However, former elite athletes are an understudied group and the existence of supportive programs is paramount to improve the quality of their post-career life [3,13]. The promotion of healthy lifestyle programs among inactive former top-level athletes with excess weight is very scarce. In the United States, within the NFL, an obesity prevention program for retired athletes was implemented under the auspice of the Living Heart Foundation and supported by academic institutions [17]. However, it is still unknown if a lifestyle intervention targeted at former athletes would be able to improve and sustain healthy lifestyle behaviors, such as increased PA, reduced sedentary behaviors, and better dietary choices.

Moreover, and although lifestyle interventions (including diet and exercise) are recognized as a non-pharmacological approach to prevent and treat obesity, many participants are not successful, or only modestly [18]. This might be due to physiological and neurohormonal adaptations in response to weight loss and/or poor adherence rates. After diet and/or exercise-induced weight loss, our body tends to retake the “set point” [19], leading to behavioral and/or metabolic compensatory mechanisms, which may be the cause of short and long-term weight management difficulties [20]. Some of these mechanisms include compensatory changes in EE [20], spontaneous PA [21], and adaptive thermogenesis [22,23,24,25]. Compensatory changes in EI have also been reported [22], and seem to be determined by exercise-induced alterations in the explicit/implicit reward value of foods, which in turn affect food choices [26]. Also, these compensatory responses may result from psychological mechanisms underpinning the interplay between exercise and eating behaviors, namely the type of motivations that guide these behaviors [27,28]. A great body of research, under the Self-Determination Theory (SDT) [29,30,31], has shown that behaviors that are adopted with a sense of choice and volition (i.e., personally endorsed). Hence, behaviors fueled by autonomous motivation, as opposed to behaviors adopted to comply or obtain others’ approval (i.e., controlled motivation), lead to lasting lifestyle changes and more favorable weight management outcomes [32,33,34]. Therefore, understanding how energy balance (EB) is regulated under lifestyle interventions that are expected to induce weight loss is paramount for developing strategies that ensure participants’ success.

In this regard, interventions that support autonomous motivation for physical activity may foster increased engagement in self-regulation techniques and positively affect lifestyle health behaviors [35]. A systematic review on effective behavior change interventions designed to promote PA and healthy eating [36] clearly supported the inclusion of multiple self-regulatory techniques, specifically self-monitoring of behavior in combination with prompt intention formation, prompting specific goal setting, providing feedback on performance, and prompting review of behavioral goals [37]. Therefore, the inclusion of self-regulatory skills within autonomy-supportive educational sessions brings added value to encourage the adoption of energy balance behaviors.

This paper describes the protocol for a randomized controlled trial, which aims to evaluate the effectiveness of the Champ4Life program in supporting former athletes to improve their lifestyles versus a waiting list control group that is offered the program after the 1-year follow-up. The primary aim of the trial is to determine whether Champ4Life can help inactive former athletes aged 18–65 years with a body mass index (BMI) ≥25 kg/m^2^ to reduce total and abdominal fat mass over 12 months. Secondary outcomes include cardio-metabolic blood biomarkers (e.g., glucose, insulin, HbA1c, lipids, liver function), adaptive thermogenesis blood-derived indices (leptin and thyroid function), weight and waist circumference, improvement in other body components (fat-free mass, water compartments), resting systolic and diastolic blood pressure, physical fitness (e.g., cardiorespiratory fitness, strength), free-living PA, sedentary time, energy balance regulation, eating self-regulation markers, and quality of life.

## 2. Methods

### 2.1. Study Design

**Figure 1:**
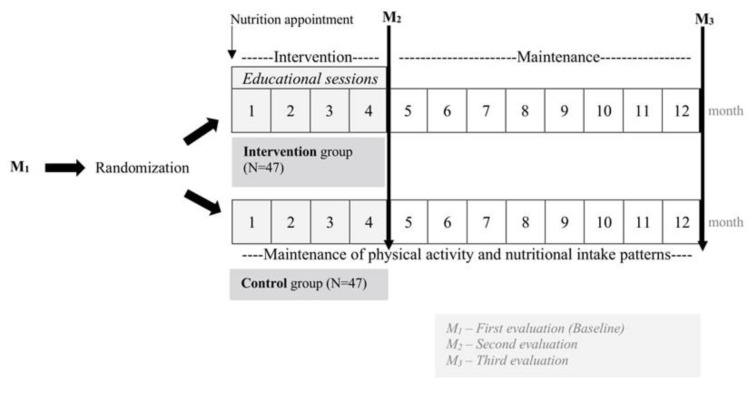
Schematic description of the study phases.

Champ4life is a 1-year randomized controlled intervention program and will be performed among healthy former top-level athletes (i.e., having represented Portugal at least once in international sport championships, or been professional soccer players in the first divisions of the championships), with overweight and obesity. The intervention and all the measurements will take place at the Exercise and Health Laboratory in the Faculty of Human Kinetics, University of Lisbon (FMH-UL). A schematic description of the study phases is presented in Figure 1.

Before enrolling in the study, participants will perform baseline assessments of body composition, free-living PA, resting energy expenditure (REE), blood pressure, physical fitness, metabolic markers, eating self-regulation, energy balance components, and quality of life. All evaluations will be performed in three sessions, starting at 8:00 am in the morning after an overnight fast, with the exception of the physical fitness tests, physical activity monitoring, food recording and the quality of life questionnaire. A schematic description of all the measurements is presented in Table 1.

The study was approved by the Ethics Committee of the Faculty of Human Kinetics, University of Lisbon (Lisbon, Portugal) (CEFMH Approval Number: 16/2016) and will be conducted in accordance to the declaration of Helsinki for human studies from the World Medical Association [38]. It has been registered at www.clinicaltrials.gov (clinicaltrials.gov ID: NCT03031951) prior to participants’ recruitment.

### 2.2. Sample Recruitment and Selection

A total of 94 healthy participants of both genders will be selected. The Portuguese Olympic Committee, the Union of Professional Soccer Players, and several national sports federations (Portuguese Judo Federation, Portuguese Swimming Federation, Portuguese Football Federation, and Portuguese Wrestling Federation) are partners of this project, and will able to recruiting former top-level athletes through direct mail, databases, and referral sources provided by these institutions. Media advertising is also planned. Brochures and posters summarizing the goals of the study, procedures, and measures to ensure participant safety will be created. Following an initial web and/or telephone screen to determine the eligibility of potential participants, an orientation session will be scheduled to offer detailed information about the study will be provided, including the number and type of assessments, the length and nature of the exercise training and the time commitment required to complete the study. The eligibility criteria are described in Table 2. A “yes” or “no” question was used to evaluate if participants did not report any exclusion criteria.

Next, potential participants will undergo run-in visits meant to reduce attrition by allowing them to determine if they will be able to schedule the study into their weekly routine. All run-in sessions must be completed within a 1-week period to satisfy the eligibility criteria. During the screening, staff members will verify that eligible participants are not currently meeting public health PA guidelines and obtain their informed consent to participate in the study.

### 2.3. Screening Process

The purpose of the staged screening process is to (1) verify eligibility; (2) obtain the participant’s informed consent; (3) complete baseline measurements; and (4) perform randomization.

The study will include several screening steps before randomization:

- A preliminary screen will be completed to determine eligibility either by phone or at the FMH-UL in separate visits to eliminate a substantial proportion of ineligible volunteers;
- Debriefing session for potential participants will be provided at FMH-UL with information about the study, including clarification of possible questions;
- Visit 1, taking place at FMH-UL, will consist of a physician appointment and a first informed consent form required for laboratory visit 1. It should be completed within 1 week after the debriefing session;
- Laboratory visit 1 will be required for participants to use accelerometers for PA assessment, individual calibration of motion sensors, and to fill dietary records (1 week). Successful completion will be required for eligibility. A second informed consent will be obtained for visits 2 and 3, which must be completed within 1 week;
- Laboratory visits 2 and 3 will include body composition, resting energy expenditure, physical fitness measurements, blood sample collection, eating-related measurements, and the completion of an exercise motivation questionnaire and a quality of life questionnaire. At the end, a third informed consent will be obtained to allow program enrollment.

Flexibility and alternative approaches in scheduling these steps are expected due to varying local circumstances, but all screening procedures will take place before randomization to guarantee each participant’s eligibility. Additional screening visits may be required due to time constraint issues.

### 2.4. Randomization Visit

Randomization will be performed to allocate eligible participants to an intervention or control group, according to an automated computer-generated randomization scheme that will be controlled by the principal investigator. Randomization will be stratified by sex, and use random length blocks and sequentially numbered opaque sealed envelopes. The study will be single-blinded: assessors for all outcomes will be blinded to participant group assignment, and all outcome data will be kept blinded until the final data entry for the entire study is completed. Participants will then be booked into their first intervention session.

### 2.5. Lifestyle Intervention Program (Intervention Group)

The Champ4life program aims to promote sustained lifestyle habits on inactive former athletes with overweight/obesity through educational sessions focused on nutrition and PA.

Briefly, the intervention group will start the lifestyle intervention with an individual 60-min consultation with a certified dietitian. This meeting is intended to increase awareness of each individual’s eating pattern and how it is contributing to their excess weight. Hence, based on that information, some key areas to work on (at the self-regulation level) and a few personally adjusted dietary strategies that contribute to a moderate reduction from ~300 to 500 kcal/day will be discussed, according to each participant’s energy requirements and preferences. Two additional follow-up appointments are also planned to adjust the individual energy requirements throughout the intervention.

Subjects will attend 12 educational sessions aimed at promoting a healthy lifestyle, in groups of 10–15 participants, for approximately four months. Sessions will last 60–90 min and will include educational content and practical application in-class exercises in the areas of PA and exercise, diet and eating behavior, and behavior modification [36]. The first session named “ABC of weight loss” is an initial session aimed at discussing body composition components (body fat and fat-free mass) and how energy balance and its components (energy expenditure and energy intake) are regulated. It will also be discussed the impact of weight-sensitive sports on body weight regulation and health-related issues [40,41]. In the second session, given by a psychologist, eating behavior dysregulation, metabolic and behavioral compensatory mechanisms will be discussed and strategies to distinguish real from “emotional” hunger and emotional eating will be the central topics. In the 3rd session, basic nutrition concepts such as the three macronutrients (protein, lipids and carbohydrates), vitamins and minerals will be addressed. We will also discuss the food wheel based on the Mediterranean diet and the healthy eating pyramid. In the fourth session, the benefits of being active will be the main topic and some recommendations will be given in order to increase daily routine PA and structured physical activity. The fifth session will be the extension of Session 2 and a dietitian will present several types of diets, its pros and cons, and lead a discussion/reflexion about which diet is the best for each type of participant. Sedentary behavior will be the main topic of the sixth session, presenting strategies to reduce sedentarism. On the seventh and eighth sessions, the pros and cons of fasting, meal frequency and its influence on weight loss, and which foods are more adequate to increase satiety in order to reduce weight, will be addressed. The next session will focus on food labels, teaching participants how to properly read and understand them, namely the ingredient lists and the nutritional composition tables (macro- and micronutrients). The tenth and 11th sessions will address the differences between several cooking methods and teach participants about the best method to decrease fat content and the best replacements to create low-calorie meals, which enhance their weight loss. In the last session, a dietitian will provide strategies to foster weight loss maintenance and a healthy lifestyle.

During the sessions, positive interactions will be encouraged in order to build fruitful relationships among participants. Positive and supportive social connections will be encouraged through interactions in social media platforms (i.e., WhatsApp group^®^ and Facebook^®^). These interactions will allow the sharing of experiences (i.e., common difficulties and possible solutions) regarding changes in PA and/or food habits amongst participants, and between participants and the program team. Support will be convened when necessary throughout the intervention. These interactions will be aimed at helping participants to embed the new behaviors into their everyday life, in order to maintain these changes in the long term.

The promotion of an autonomy-supportive intervention climate, facilitating long-term integration of new health behaviors, and prompting self-regulation skills such as self-monitoring of behavior through pedometer use, regular food records, and self-weighing, will also be a central part of the curriculum (present in all sessions and contents). Indeed, the program is theoretically based on SDT [29,30,31], which postulates that human beings have essential psychological needs for autonomy (feeling of being the origin of one’s own behaviors), competence (feeling effective), and relatedness (feeling understood and cared for by others). These needs represent “psychological nutriments”, and their support and subsequent satisfaction provide the basis for autonomous motivation, essential for the initiation and long-term maintenance of health behaviors [30,32]. Thus, several strategies will be developed to support these basic psychological needs, using several behavior change techniques and self-regulatory skills: (i) encouragement of self-selected relevant goals (for weight, PA and eating); (ii) encouragement of volitional (not compulsory), regular self-weighting, and self-monitoring of PA and eating behaviors. In this regard, a pedometer will be given to all participants and they will be encouraged to record their daily steps and keep weekly records. A pocket-worn activity monitor is an effective strategy to improve PA behavior [36] and has been shown to be a useful tool in other programs [42]. To facilitate the record, an excel file with formulas embedded to present the % of increase and decrease, facilitating the sense of progression, will be provided. Metrics for success according to international guidelines will be given, but the focus will be on individual progress (e.g., achieving 10,000 daily steps is important, but the goal will be set in terms of raising one’s personal level of steps per day). Increases will be celebrated as successes using short-term goals towards the long-term goal (i.e., reaching the internationally recommended levels). The above-mentioned self-monitoring (PA, weight and eating) will be used to raise self-awareness of one’s patterns and also to identify barriers and facilitators of the desired changes. (iii) Providing rational for change will be achieved by prompting key information regarding the benefits and consequences of one’s choices (PA and eating), as well as basic knowledge allowing informed decisions and choices. For example, to assist food shopping at the supermarket, a nutritional card developed by our National Health Administration System (https://nutrimento.pt/activeapp/wp-content/uploads/2015/11/Cart%c3%a3o-Rotulos-para-impress%c3%a3o.pdf), using a color system will be given. Participants will be encouraged to make small but enduring reductions in energy intake and to increase energy expenditure to induce a daily energy deficit of approximately 300 kcal. In the first session, participants will be informed that reaching a minimum of 3–5% weight loss at four months is likely to result in clinically meaningful health benefits, as per international guidelines [43], and will be subsequently prompted to calculate the number of kg that corresponds to it in their particular case.

Positive feedback and celebration of individual progresses (by making short-term goals) will be given in order to help participants feel competent and confident about their long-term PA, sedentary behavior, weight loss, and healthy eating targets [44].

A detailed session-by-session manual will be also developed and participants will be able to track all the information provided at the weekly sessions.

### 2.6. Waiting List (Control Group)

The control group will be placed on a waitlist to be offered the Champ4Life program after completing the 12-month follow-up measurements. In addition, this group will receive multimedia health information fortnightly by e-mail over the first 12-month period. The information provided will cover general healthy lifestyle topics, including tips for healthier dietary habits and to avoid excessive alcohol consumption and tobacco use.

### 2.7. Adherence Promotion Efforts

Retention will be promoted through a variety of methods, namely promoting regular contact with all participants, assuring attendance to the sessions, and providing social support. The focus will be on identifying and addressing participants’ concerns before they express a desire to reduce their involvement in the study.

#### 2.7.1. Strategies to Engage Participants Avoiding Low Attendance

The investigators will seek to maintain regular contact with participants (e.g., phone, e-mail, WhatsApp^®^ group) via positive social interactions. For example, to stimulate interest and increase session attendance, prior to each educational session, a brief summary of the highlights of the session will be sent via WhatsApp^®®^ to increase session attendance and participation. Support materials for each session will be also be sent. Given the inter-connectedness between all session contents, participants will be encouraged to attend all sessions. When/if a participant starts skipping several sessions, the team will express interest in understanding what may not be working for that participant. The importance of attending assessment visits will also be highlighted, given the experimental nature of the study. Participants will be encouraged to understand the importance of the assessments per se (for the advancement of science) despite their success/unsuccess in the intervention (to preclude differential/biased attrition rate). All will be viewed as important and crucial for the study.

#### 2.7.2. Rewards for Participation

Randomized participants will not receive any type of financial incentive, although all will receive the lifestyle intervention program and manual (the control group will receive it after completing the 12-month assessments).

## 3. Measurements

### 3.1. Body Composition

#### 3.1.1. Anthropometry

Subjects will have their weight and height measured in bathing suits and no shoes to the nearest 0.01 kg and 0.1 cm, respectively, with a stadiometer (Seca, Hamburg, Germany). Body mass index will be calculated using the formula [weight(kg)/height^2^(m^2^)] and the cut-off points of the World Health Organization (WHO) will be used [45]. Waist circumference will be measured immediately above the iliac crest and reference values proposed by WHO will be used [46]. Triceps, subscapular, abdominal, supra-iliac, thigh, and calf skinfolds and arm, thigh, and calf circumferences were assessed by a technician accredited by ISAK (International Society for the Advancement of Kinanthropometry), as described elsewhere [47].

#### 3.1.2. Dual-Energy X-ray Absorptiometry

To estimate total and regional fat mass (FM) and fat-free mass (FFM), dual-energy X-ray absorptiometry (DXA; Hologic Explorer-W, Waltham, MA, USA) will be used. A whole-body scan will be performed and the attenuation of X-rays pulsed between 70 and 140 kV synchronously with the line frequency for each pixel of the scanned image will be measured. Total abdominal fat, which includes intra-abdominal fat plus subcutaneous fat, will be distinguished using DXA by identifying a specific region of interest (ROI) within the analysis program. Specific DXA ROIs for abdominal regional fat will be defined as follows: from ROI 1, the upper edge of the second lumbar vertebra (approximately 10 cm above the L4 to L5) to above the iliac crest and laterally encompasses the entire breadth of the abdomen, thus determining total abdominal fat mass [48].

#### 3.1.3. Bioimpedance Analysis (BIA)

Before the test, subjects will be instructed to lie in a supine position with their arms and legs abducted at a 45 angle for 10 min. The measurements assessed by the bioimpedance devices (described below) will be performed in a random order (time difference 30 s). Four electrodes will be placed on the dorsal surfaces of the right foot and ankle and the right wrist and hand. A 240 μARMS alternating current at 50 kHz will be introduced into the distal electrode of each pair (source electrode), and the voltage drop across the body will be measured using the proximal electrode (detector electrode). Low-impedance electrodes (25.74 Ω) (AKERN-BIATRODES/0ELB) will be used in both multispectral and single frequency devices.

#### 3.1.4. Multispectral Frequency Bioelectrical Impedance

Bioelectrical impedance spectroscopy analysis (BIA; Xitron Technologies model 4200 B, San Diego, CA, USA) will be used to assess total-body water (TBW) and its compartments. This impedance spectra is modelled with the Cole–Cole cell suspension model [49] to derive a theoretical impedance at zero and infinity frequency, based on a non-linear curve fitting from the measured resistance and reactance. Intracellular water and extracellular water will be predicted from the Hanai mixture theory [50], and TBW will be estimated by the sum of intracellular water and extracellular water.

#### 3.1.5. Single-Frequency Bioelectrical Impedance

Whole body R, Xc, and phase angle will also obtained by BIA using a single-frequency, phase-sensitive 50 kHz (BIA-101, RJL/Akern Systems, Firenze, Italy). Prior to each test, the technical validity of SF-BIA instrument will be determined with a precision circuit (R = 383 Ω and capacitance = 46 Ω). Measured resistance and reactance values will be within the tolerance of the precision circuit (≤10 Ω and ≤5 Ω, respectively).

#### 3.1.6. Resting Energy Expenditure

Assessment of REE will be performed in the morning when fasted (8.00–10.00 a.m.). All measurements will be performed in the same room at environmental temperature and humidity of approximately 22 °C and 40–50%, respectively. The MedGraphics CPX Ultima (MedGraphics Corporation, Breezeex Software) indirect calorimeter will be used to measure breath-by-breath oxygen consumption ($\dot{V}O_{2}$) and carbon dioxide production ($\dot{V}CO_{2}$) using a facial mask. One trained technician will conduct all measurements. The oxygen and carbon dioxide analyzers will be calibrated in the morning before testing, using known gas concentration. The flow and volume will be measured using a pneumotachograph calibrated with a 3L-syringe (Hans Rudolph, inc.TM). Before testing, participants will be instructed about all the procedures and asked to relax, breathe normally, not to sleep, and not to talk during the evaluation. Total rest duration will be 45 min, participants will lie supine for 15 min covered with a blanket and the calorimeter device will then be attached to the mask and breath-by-breath VO_2_ and VCO_2_ will be measured for a 30-min period.

The first and the last 5 min of data collection will be discarded and the mean of a 5-min steady state interval between the 5 and 25 min with a respiratory exchange ratio (RER) between 0.7 and 1.0 will be used to determine REE. Steady state will be defined as a 5-min period with ≤10% CV for $\dot{V}O_{2}$ and $\dot{V}CO_{2}$ [51]. The mean $\dot{V}O_{2}$ and $\dot{V}CO_{2}$ of 5-min steady states will be used in the Weir equation [52] and the period with the lowest REE will be considered for data analysis.

#### 3.1.7. Adaptive Thermogenesis

In order to predict REE at baseline, a gender-specific regression equation using FFM and FM as the independent predictors will be generated. This equation will then be used to predict REE at four months and after a 12-month follow-up, using the measured values of FM and FFM at these time points. To disclose any adaptations in REE not accounted for by changes in FFM, the adaptive response will be assessed as 1 min the ratio between the actual to the expected/predicted REE, multiplied by 100. Positive values indicate a decrease in REE beyond that expected by changes in body composition (actual REE below predicted REE) whereas negative values represent a change in REE equal to or greater than the predicted REE (actual REE higher than the predicted REE) [20]. The magnitude of the adaptive thermogenesis will be further analyzed by examining leptin levels and thyroid function.

#### 3.1.8. Energy Intake

When total energy expenditure (TEE) is obtained in conjunction with measured body composition, then two terms of the EB equation are known, namely EB and energy expenditure (EE). From the previous equation, we can estimate average energy intake during the course of the study. The magnitude of compensation will be calculated as the difference between achieved energy imbalance and EE. Three-day food records (with one-weekend day) will also be collected to characterize macronutrient composition of the diet in the three assessment sessions using a software package (Food Processor SQL, ESHA Research, Salem, OR, USA) by a registered dietitian. Comprehensive written instructions using specific guidelines, including pictures of portion sizes used for a better recording of food intake, and examples of common errors in recording dietary intake combined with face-to-face debriefing sessions will ensure accurate food records collection. At the end of the recording period, a registered dietitian will review the record with the participant to clarify potential omissions and ambiguities and to assure that additional information is provided to improve the accuracy of the macronutrient composition of the diet.

### 3.2. Free-Living Physical Activity and Energy Expenditure

#### 3.2.1. Accelerometry

All participants will be asked to wear an accelerometer ActiGraph GT3X+ (ActiGraph, Pensacola, FL) to assess the amount of activity expressed as minutes per day spent in different intensities. The accelerometer will be placed on the right hip, near the iliac crest, during waking-hours, and requested to be removed only during water-based activities such as showering and swimming [53]. The device activation, download, and processing will be performed using the software Actilife (v.6.9.1). The cutoff values used to define the intensity of PA and therefore to quantify the mean time in each intensity (sedentary, light, moderate, or vigorous) will be as follows: sedentary: <100 counts·min^−1^; light: 100–2019 counts·min^−1^; moderate: 2020–5998 counts·min^−1^ (corresponding to 3–5.9 METs); vigorous: ≥5999 counts·min^−1^ (corresponding to ≥6 METs) [54]. Participants will be included if they had a total of at least three valid days of accelerometer data. A valid-day will be defined as having 600 or more minutes (≥10 h) of monitor wearing, corresponding to the minimum daily use of the accelerometer [55]. Apart from accelerometer non-wearing-time (i.e., when it is removed for sleeping or water activities), periods of at least 60 consecutive minutes of zero activity intensity counts will also be considered as non-wearing-time [56]. An Actigraph break will be considered as any bout of time in which the accelerometer count rises up to or above 100 counts·min^−1^ and which stays within the light-intensity physical activity (LIPA) range (<2020 counts·min^−1^). There are no cutoffs for the sedentary-time using the three-axial information from this new generation Actigraph GT3X+ accelerometer; therefore, we will use the previous cutoffs, which utilize the vertical-axis information only.

#### 3.2.2. Combined Heart Rate and Motion Sensor

The Actiheart (Actiheart, CamNtech Limited, UK) is a lightweight (10 g) combined heart rate (HR) and movement (uniaxial accelerometer oriented to measure acceleration along the body’s longitudinal axis) sensor that utilizes both piezoelectric accelerometer and HR data synchronously. An individual calibration using a standardized step test will be realized. This test consists of step up and down a 15-cm high step, progressively increasing step frequency from 15 to 32.5 body lifts per minute (rate of change: 2.5 body lifts per min^2^). If some of the participants are not able to finish the step test, we will perform a group calibration that is available in the software (version 4.0.99). The Actiheart will be started in the long-term mode to record HR and acceleration with 15 s epochs. Participants will wear the Actiheart 24 h day−1 at the same time as the accelerometer and a valid day will be defined as having 600 or more minutes (≥10 h) of monitor wear during waking hours. Data from the Actiheart will be downloaded into the commercial software. The camNtech software algorithm allows data cleaning, recovering, and interpolation of missing and noisy HR. Using the raw data from the branched combined model that uses activity (acceleration) and HR, it is possible to extract and quantify the daily sedentary time (<1.5 METs). A break in sedentary time will be considered whenever participants are above the aforementioned cut-offs. Participants will also be instructed to register the periods in which they remove the device for water activities.

TEE will be predicted using the Crouter et al. equations [57]. Physical activity energy expenditure (PAEE) will be calculated as TEE minus (0.1 × TEE + REE), assuming the thermic effect of food represents 10% of TEE.

The delivery and fitting of the device to the participants will be conducted face-to-face [55]. The devices will be activated on the first day at 6.00 a.m. and data will be recorded in 15-sec epochs. Participants will be asked to record timing and reasons for every occasion the devices are removed.

#### 3.2.3. Eating Self-Regulation Markers

The reward value of food (i.e., implicit wanting/explicit liking and wanting) will be measured by a computer procedure, the Leeds Food Preference Questionnaire (LFPQ) [58,59], which uses a “forced choice” reaction time measure of implicit wanting in addition to explicit subjective measures of liking and wanting for visual food stimuli varying in fat content and taste. To measure implicit wanting, the question “Which food do you most want to eat right now?” will be presented and the participant will be required to choose between a pair of food images as quickly as possible (with minimum thinking). The speed with which one category of stimuli is chosen relative to alternative categories provides a quantifiable measure of implicit wanting for each food category in the procedure. Reaction times as well as frequency of choice will be recorded. Explicit liking and wanting measures will be obtained by rating the same stimuli according to a 100-mm visual analogue scale (VAS), with a total of 16 food images, after prompting the subjects with the statements ‘‘How pleasant would it be to experience a mouthful of this food now?’’ for explicit liking and ‘‘How much do you want some of this food now?’’ for explicit wanting. For both scales, the anchor “Not at all” will be used on the left side of the scale and “Extremely” on the right side. Both questions will be presented intermittently and in a random order. Finlayson et al. showed that this questionnaire is sensitive to changes in the physiological state induced by eating and exercise, which suggests that it is suitable for capturing the changes in food reward when a change in individual’s state occurs [58,60]. It has been previously used in individuals with overweight and obesity, as well as in anti-obesity drug trials [61,62].

Intuitive eating and motivations to regulate eating will be assessed using internationally validated questionnaires, namely the Intuitive Eating Scale—2 (IES-2) [63] and the Regulation of Eating Behavior Scale (REBS) [64]. IES-2 is a 23-item questionnaire that measures the degree to which one eats in response to physiological eating cues, comprising 4 subscales: eating for physical rather than emotional reasons (Cronbach’s α = 0.92), unconditional permission to eat (Cronbach’s α = 0.81), reliance on hunger and satiety cues (Cronbach’s α = 0.85), and body-food choice congruence (Cronbach’s α = 0.83) [63]. Participants respond to the stem “For each item, please check the answer that best characterizes your eating attitudes or behaviors” on a 5-point Likert scale ranging from 1 (“strongly disagree”) to 5 (“strongly agree”). REBS comprises a total of 24 items, organized into 6 subscales, measuring amotivation, external, introjected, identified, integrated, and intrinsic motivations. Following the stem “Why are you regulating your eating behaviors?”, participants respond on a 7-point Likert scale ranging from 1 (“does not correspond at all”) to 7 (“corresponds exactly”). Prior research has supported REBS’s factor structure and reliability (Cronbach’s alphas >0.70) [64].

#### 3.2.4. Blood Samples

Measurement of glucose, lipid profile, including total cholesterol, low (LDL) and high-density lipoprotein (HDL), cortisol and hepatic enzymes will be performed in serum samples using colored enzymatic tests, in an automated analyzer (Cobas Integra 400). Insulin assessment will be performed in an automated analyzer (Cobas Integra 400) by eletroquimioluminescence. HbA1c will assessed by high-performance liquid chromatography. High sensitive C-reactive protein will be determined by nephelometry. Homeostatic model assessment for insulin resistance (HOMA-IR), a procedure to quantify insulin resistance, will be calculated through the following equation [65,66]:

$$HOMA-IR=\;\frac{Fasting\;Insulin\;\left(\mu U/mL\right)\;\times \;Fasting\;Glycemia\;\left(mmol/L\right)\;}{22.5}$$

Reference values for cholesterol and its components [67], *Glycemia* [68] and thyroid panel [69] will be considered.

Metabolic syndrome (MetSyn) may be present whenever at least 3 of the 5 following risk factors are present in a participant: (a) abdominal obesity, characterized by an abdominal circumference higher than 102 and 88 cm for men and women, respectively; (b) hypertriglyceridemia, that is values of triglycerides equal or higher than 150 mg/dL; (c) low HDL cholesterol, identified by values of HDL cholesterol equal or below 40 or 50 mg/dL for men or women, respectively; (d) high arterial blood pressure, when systolic and diastolic arterial blood pressure are equal or higher than 135 and 85 mmHg, respectively; (e) hyperglycaemia, that is fasted glucose equal or higher than 100 mg/dL [70].

Blood derived-indices will also include the assessment of the thyroid panel (T3, T4, TSH) by immunoassay with chemiluminescent detection (Millipore Corp., Billerica, MA, USA,) and leptin levels by ELISA (enzyme-Linked Immunosorbent Assay).

### 3.3. Physical Fitness

#### 3.3.1. Cardiorespiratory Fitness Test

Maximal cardiorespiratory capacity will be assessed using the Bruce protocol test [71] performed on a variable speed and incline treadmill (Quinton Treadmill, Model 640, 90 TM Series). Simultaneously, the ventilated gas volumes, flow rates, and respiratory gas exchange will be determined by an open-circuit spirometry system (MedGraphics Corporation, Breezeex Software). Maximal oxygen uptake (VO_2_max) will be achieved when the two following criteria are obtained: a respiratory exchange ratio equal or greater than 1.10 for females and males aged to 20–49 y and equal or greater than 1.05 for females and males aged to 50–64 y [72], and no increase in VO_2_max despite further grade increases. The assessment will be performed at baseline and at 12 months.

#### 3.3.2. Strength Tests

The handgrip strength test evaluates the maximal isometric force of the muscles of the hand and forearm and will be assessed by a portable hand dynamometer JAMAR plus digital (Sammons Preston, Bolingbrook, IL, USA). Participants will be assessed on both hands alternately, in a standing position. Prior to the test, the grip dynamometer will be adjusted to the size of the hand of each subject. Handgrip assessment will be conducted with the subject standing up with the arms in a neutral position (halfway between supine and pronation position). Each participant will be assessed on both hands alternately until reaching three attempts for each hand. In each attempt, the subject will exert the maximal grip strength on the handgrip dynamometer with the assessed hand for 5 s. After each attempt, there will be a resting period of 60 s that will be used both for recovery and for changing the handgrip dynamometer to the opposite hand. The assessment is performed at baseline and at 12 months.

For the lower body strength, we will use the maximum strength of the leg by performing a horizontal leg press isometric test (S0409, BPH) with the bent leg and the knee joint at an angle of 110°. Participants will complete 5 maximal voluntary repetitions lasting 30 s, and a period of rest between reps of 60 s. All participants will be asked to produce strength the fastest they possibly can in all repetitions. The Plux software (Biosignalsplux) will be considered to analyze the highest value between the maximal voluntary repetitions. All participants will be instructed not to perform a Valsalva manoeuvre during the tests. The assessment will be performed at baseline and at 12 months.

#### 3.3.3. Resting Systolic and Diastolic Blood Pressure

Three measurements of systolic and diastolic blood pressure will be obtained with the participant in the sitting position using a digital sphygmomanometer. The blood pressure cuff-fixed on the nondominant upper arm is loosened during the two min pause between the measurements. The arithmetic means of the second and third readings are considered as the participants’ blood pressure and pulse values, respectively. Overall, there is a rest of approximately five minutes before the second blood pressure measurement is carried out. The cut-off values of 140 mmHg for systolic blood pressure and 90 mmHg for diastolic blood pressure will be considered [73].

#### 3.3.4. Exercise Motivation

The Behavioral Regulation in Exercise Questionnaire—3 (BREQ-3) [74,75] will be used to assess motivations to engage in exercise. BREQ-3 contains 24 items, organized into 6 subscales, measuring external, introjected (i.e controlled motivational regulations), identified, integrated, and intrinsic motivations (i.e, autonomous motivational regulations). Following the item “why do you exercise”, participants respond on a 5-point Likert scale ranging from 0 (“not true for me”) to 4 (“very true for me”). Prior research has supported BREQ’s factor structure and reliability (Cronbach’s alphas >0.70) [74,75,76].

#### 3.3.5. Quality of Life

To measure general health-related quality of life, participants will complete the Short-Form Health Survey (SF-36) questionnaire [77], with a total of 36 items composed of eight dimensions: physical functioning (Cronbach’s α = 0.83), physical role limitations (Cronbach’s α = 0.89), bodily pain (Cronbach’s α = 0.88), general health (Cronbach’s α = 0.82), emotional role limitations (Cronbach’s α = 0.74), social functioning (Cronbach’s α = 0.71), vitality (Cronbach’s α = 0.86), and mental health (Cronbach’s α = 0.90) [77]. These dimension scores will be summarized into two composite scales, a physical component summary and a psychological/mental component summary. Statements such as ‘‘During the past 4 weeks, I cut down the amount of time I spent on work or other activities’’ will be answered with yes or no; questions such as ‘‘How much bodily pain have you had during the past 4 weeks?’’ will be evaluated on a 6-point Likert scale from none to very severe. Higher scores indicate better health-related quality of life.

#### 3.3.6. Statistics

Statistical analysis will be performed using IBM SPSS statistics version 24.0 (IBM, Chicago, IL, USA). To test the normality of distribution of the variables, the Kolmogorov–Smirnov test will be performed. Descriptive statistics will be calculated (mean, standard deviation, and range). If normality is not achieved in some variables, non-parametric methods will be used. Repeated measures with Bonferroni corrections for adjusted comparisons will be used to determine any differences between conditions at the three moments and for changes between the intervention and control groups over time in primary and secondary outcomes. For exploring the association between changes in body composition and metabolic parameters, linear mixed regression models will be conducted. Statistical significance will be set at *p* < 0.05.

#### 3.3.7. Sample Size

For sample and power calculations, this study is powered on changes in total body fat assessed by DXA. We consider a type I error of 5% and a power of 80% (using the software GPower version 3.1.9.2) to detect an effect size of 0.58 for statistically significant differences in total body fat as reported elsewhere [78]. A total of 37 participants per group will be required. Considering a 20% drop out rate, as occurred in the selected study [78], we will enroll 94 participants (47 in each group).

## 4. Discussions

This paper describes the protocol of the Champ4life project, a study that aims to develop an effective lifestyle intervention for former athletes with overweight and obesity.

The ending of a sports career is inevitable, and athletes are often faced with challenges to adapt to their new lifestyle. It is expected that when an athlete reduces his/her PAEE, an equivalent reduction in his/her EI occurs. However, the current literature shows that a sedentary routine does not induce a compensatory reduction in E, showing that former athletes will likely feel challenged in the adaptation to their new reality as regards to their PA and food choices routines. The effect of a lifestyle intervention on former athletes that have become inactive and gained excessive weight is still understudied.

This will be the first randomized clinical trial spanning a one-year intervention period aimed at understanding the effects of a lifestyle intervention on multiple health outcomes in former athletes. By empowering participants with key knowledge, forming the basis for informed decisions, and most of all, with a set of central self-regulatory skills (self-monitoring, action planning, and goal setting), it is expected that there will be an effective promotion of sustained improvements in body composition (especially by reducing total and abdominal FM), metabolic markers, health-related quality of life [79], and physical function [80].

According to the Endocrine Society, only a low percentage of people who lose weight (<20%) are able to maintain it [81], despite the effectiveness of common strategies in the short-term. In fact, the first year after the weight loss is the most challenging, with one-third of the lost weight tending to be regained over that year [82].

Two years after weight loss, 40% of the people who achieved a modest weight loss (at least 5% of initial weight), regained at least half of their lost weight during the maintenance period [81]. The current program is based on SDT, which has been previously proven effective in the promotion of long term behavioral changes and weight-loss [30,32,83]. This program will focus on the promotion of participant’s autonomous motivation towards their weight management process and on the provision of useful knowledge and tools to help them sustain their healthy behaviors. Therefore, successful maintenance of the positive lifestyle changes and lost weight is expected.

In addition, prior research has shown an increase in upper and lower body strength after diet and exercise programs [84]. Other studies have shown that after lifestyle interventions, an increase of cardiorespiratory fitness occurs [84,85,86]. Therefore, it is expected that after the Champ4life intervention, participants reveal an increase in physical fitness, leading to higher muscle quality, better cardiorespiratory fitness, and lower mortality [86].

We also expect to understand the metabolic and behavioral compensations that may occur during weight loss and maintenance. We aim to find any compensations which lead to the retake of initial weight, like changes in energy intake and expenditure [20], spontaneous PA [21], and adaptive thermogenesis [22]. The current literature shows that when we increase EE (by increasing PA), we may compensate by increasing sedentary behavior, decreasing lifestyle PA and/or increasing energy consumption during the rest of the day [87]. The mechanisms underlying adaptive thermogenesis (i.e, mainly the decrease in REE and PAEE), beyond what can be predicted from the changes in FM and FFM in response to a restricted EI [19], are unclear when modest weight loss is expected. The mechanisms underlying the interplay between PA and alterations in eating behaviors and dietary intake (in terms of quantity and quality) also require further exploration, in order to improve interventions’ efficacy.

A significant strength of the Champ4life program will be the SDT-based intervention, providing tools for the adoption and sustainability of healthy behaviors through the development of autonomous motivation and self-regulatory skills [30,32,83]. Some limitations are anticipated in this study and should be addressed, in particular the effect of confounding variables such as the large age range (18–65 years) of participants eligible to be included, retirement years, and an unbalanced gender representation, provided there are more male former athletes than females.

To address these issues, the analysis will be controlled for the potential effects of these confounders. In addition, the prevalence of eating disorders is higher among athletes, especially in those engaged in weight-sensitive sports [88,89], and this may also be the case with former athletes; still, this aspect might not be appropriately captured by initial screening procedures (relying on yes/no answers) and thus will constitute a limitation of the present study.

We are also expecting to find barriers in the recruitment process due to the inclusion criteria. Despite the adherence promotion efforts, we may have some difficulties in retaining the participants throughout the study, due to its duration (~1 year), in particular those randomized to the control group. To minimize this risk, the control group is, in fact, a waiting list comparison group that is offered the program after the 12-month follow-up.

In conclusion, we anticipate that Champ4Life will be a novel and unique approach to improve athletes’ healthy lifestyle in post-career life. This project will provide evidence on the effectiveness of the Champ4Life program delivered to former athletes and will offer insight on the impact of changing PA, sedentary behavior, and diet on body composition and secondary outcomes, such as EB regulation and overall health-related markers. The findings from this project will enable evidence-based decisions for the implementation of lifestyle interventions for post-career transition.

## Figures and Tables

**Table 1:** Schematic description of all measurements.

	M_1_			M_2_		M_3_		
Schedule	Visits 1 and 2 *		Visit 3	Visit 1		Visit 1		Visit 2
08:00–08:45	REE and Blood pressure	Fasted stage	Strength tests	REE and Blood pressure	Fasted stage	REE and Blood pressure	Fasted stage	Strength tests
08:45–09:00	Blood samples		Questionnaires (IES-2; REBS; BREQ-3; SF-36)	Blood samples		Blood samples		Questionnaires (IES-2; REBS; BREQ-3; SF-36)
09:00–09:20	DXA			DXA		DXA		
09:20–09:40	Bioimpedance		Cardiorespiratory fitness test	Bioimpedance		Bioimpedance		Cardiorespiratory fitness test
09:40–10:10	Anthropometry			Anthropometry		Anthropometry		
10:10–10:30	LFPQ			LFPQ		LFPQ		
10:30–10:45	BREAK			BREAK		BREAK		
10:45–11:00	Delivery of the motion sensors and food diaries with face-to-face instructions.			Delivery of the motion sensors and food diaries with face-to-face instructions.		Delivery of the motion sensors and food diaries with face-to-face instructions.		
11:00–11:45				Questionnaires (IES-2; REBS; BREQ-3; SF-36)				

Legend: Baseline (M1), Moment 2 (M2), and Moment 3 (M3); E_1_—first evaluation; E_2_—second evaluation; E_3_—third evaluation; REE—resting energy expenditure; DXA—dual-energy X-ray absorptiometry; IES-2—Intuitive Eating Scale—2; REBS—Regulation of Eating Behavior Scale; BREQ-3—Behavioral Regulation in Exercise Questionnaire—3; SF-36—Short-Form Health Survey; LFPQ—Leeds Food Preference Questionnaire. * At baseline (M1) the first visit starts with the delivering of the accelerometer, given that the physical activity level of the participants is required for assessing eligibility.

**Table 2:** Eligibility criteria.

**Inclusion Criteria**
Former high-level athlete; Aged 18–65 years (male or female); Inactive (<20 min/day of vigorous physical activity intensity for at least 3 days per week or <30 min/day of moderate intensity physical activity for at least 5 days per week [39]); With overweight/obesity (BMI ≥25 kg/m^2^); Available to participate in the educational sessions at FMH-UL; Ready to modify their diet in order to lose body weight.
**Exclusion Criteria**
Failure to complete the run-in for dietary intake and physical activity; Unable/unwilling to give informed consent or communicate with local study staff; Inability to complete the study within the designated time frame because of plans to move out of the study area; Inability to attend the visits/appointments, evaluation measurements, and attend the intervention sessions at the FMH-UL; Schizophrenia, bipolar disorder, other psychotic disorders; Eating disorders; Major depression; Current consumption of more than 14 alcoholic drinks per week or other substance abuse, and/or current acute treatment or rehabilitation program for alcohol/substance abuse; Pregnancy/planning to get pregnant within the next 8 months; Having been pregnant within the past 6 months or breastfeeding; History of weight loss surgery or liposuction procedures; Current participation in a weight loss program; Losing >4.5 kg in the past three months; Current use of medications for weight loss; Thyroid disorders; Diabetes and cardiovascular disease; Other medical conditions known to affect energy balance homeostasis; Systemic corticosteroid treatment (weight gain associated with steroids may interfere with the intervention goals but use of hormone replacement therapy or oral contraceptives will not lead to exclusion).
